# Plasma burn—mind the gap

**DOI:** 10.1098/rsta.2023.0406

**Published:** 2024-10-09

**Authors:** Hendrik Meyer, on behalf of the STEP Plasma Team

**Affiliations:** ^1^ UKAEA, Culham Campus, Abingdon, Oxon OX14 3DB, UK

**Keywords:** spherical tokamak, burning plasma, reactor, plasma scenario integration, turbulence, edge localized modes

## Abstract

The programme to design plasma scenarios for the Spherical Tokamak for Energy Production (STEP), a reactor concept aiming at net electricity production, seeks to exploit the inherent advantages of the spherical tokamak (ST) while making conservative assumptions about plasma performance. This approach is motivated by the large gap between present-day STs and future burning plasmas based on this concept. It is concluded that plasma exhaust in such a device is most likely to be manageable in a double null (DN) configuration, and that high core performance is favoured by positive triangularity (PT) plasmas with an elevated central safety factor. Based on a full technical and physics assessment of external heating and current drive (CD) systems, it was decided that the external CD is provided most effectively by microwaves. Operation with active resistive wall mode (RWM) stabilization as well as high elongation is needed for the most compact solution. The gap between existing devices and STEP is most pronounced in the area of core transport, owing to high normalized plasma pressure in the latter which changes qualitatively the nature of the turbulence controlling transport. Plugging this gap will require dedicated experiments, particularly on high-performance STs, and the development of reduced models that faithfully represent turbulent transport at high normalized pressure. Plasma scenarios in STEP will also need to be such that edge localized modes (ELMs) either do not occur or are small enough to be compatible with material lifetime limits. The high current needed for a power plant-relevant plasma leads to the unavoidable generation of high runaway electron beam current during a disruption, where novel mitigation techniques may be needed.

This article is part of the theme issue ‘Delivering Fusion Energy – The Spherical Tokamak for Energy Production (STEP)’.

## 1. Introduction

The plasma is at the heart of a fusion power plant, with its main function of generating the required neutrons that produce the heat for driving the turbine and to breed tritium from lithium in the blanket system as part of the fuel for the deuterium–tritium fusion reaction


(1.1) $$\overset{2}{\underset{1}{\;D}}+\overset{3}{\underset{1}{\;T}}\to \overset{4}{\underset{2}{\;\;\;He}}\left(3.5\;Mev\right)+\overset{1}{\underset{0}{\;\;\;\;\;\;n}}\left(14.1\;Mev\right).$$

**Figure 1.**
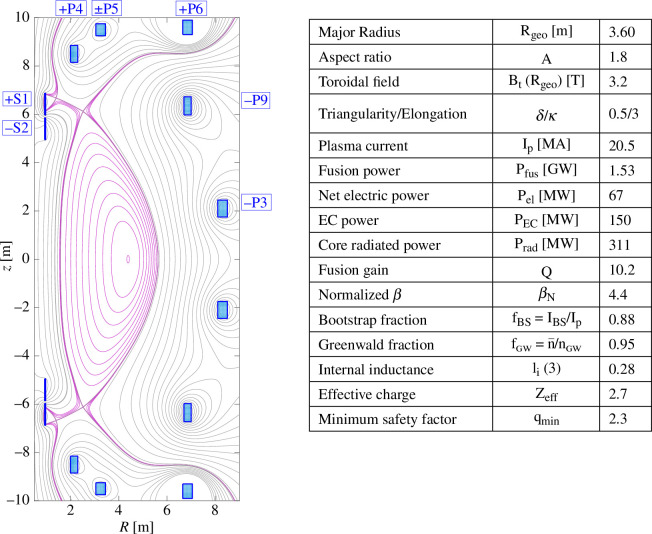
Flux contours (pink) of the equilibrium for a high-density EC wave resonance only heated FTOP with a table of respective plasma parameters. The poloidal field-shaping coils are shown in blue (filled boxes).

The 3.5 MeV ^4^He nuclei ( α -particles) generated in the deuterium–tritium fusion reaction are used to maintain the high temperature of the plasma to enable the fusion reaction to continue. A fusion power plant requires a burning plasma where the heating from the α -particles dominates over the auxiliary heating with a fusion gain $Q=\frac{P_{\text{fus}}}{P_{\text{aux}}}\gg 1$ . This is needed to generate net electricity since most of the power produced in the fusion reaction is either needed to maintain the burning plasma steady state or is lost owing to inefficiency. Typically, net electricity requires a minimum value of *Q* in the range 5–10 and a commercial power plant needs Q>30 . The Spherical Tokamak for Energy Production (STEP) prototype power plant (SPP) aims for a Q≲10 to produce $P_{\text{net}}∼100\;MW$ with a fusion power in the range of $1.5\;GW\leq P_{\text{fus}}\leq 1.8\;GW$ in a device with a geometric major radius $R_{\text{geo}}=3.6\;\text{m}$ , aspect ratio A=1.8 , toroidal field on the geometric axis $B_{t0}=3.2T$ , plasma current $I_{\text{p}}\approx 20$ –25 MA, auxiliary heating and current drive (HCD) power $P_{\text{HCD}}\approx$ 50–150 MW and high elongation κ≈3 and triangularity δ≈0.5 in the current design iteration [1,2]. Typical parameters for a specific design point are shown in figure 1.

To maximize the fusion yield the plasma needs to be confined for long enough at the highest possible density $n_{i}$ to fulfil the extended Lawson criterion (triple product) $n_{i}\tau_{E}T_{i}\;\geq 3\times 10^{21}\;keVs{\text{m}}^{\text{-3}}$ where $\tau_{E}$ is energy confinement time [3]. For typical observed scaling laws of the tokamak energy confinement time, the optimum ion temperature for break-even ( Q=1) $T_{i}\approx 15\;keV$ . Nested magnetic flux surfaces in a toroidal geometry provide one of the most promising concepts for confining such a hot plasma. The axisymmetric tokamak featuring a diverted plasma is the most advanced of these magnetic confinement concepts. Its cost is strongly driven by the volume and magnitude of the toroidal field $B_{t}$ needed to confine a plasma with a given pressure p and the size of the device [4]. The dimensionless quantity $\beta_{t}=2\mu_{0}\langle p\rangle /B_{t0}^{2}$ (where angled brackets indicate volume averages), measuring the confined plasma pressure normalized to magnetic pressure, is often used as a cost indicator with a higher $\beta_{t}$ likely leading to more cost-effective plants as $P_{\text{fus}}/V\propto p^{2}\propto \beta^{2}B_{t}^{4}$ even though a direct scaling is difficult to ascertain [4]. More details on costing can be found in ‘Optimising the cost of the STEP programme’ in the same issue [5].

STs, the concept on which STEP is based, with a tight aspect ratio $1.3\leq A=\frac{R}{a}\leq 2$ ( R,a : major and minor radii of the torus) have achieved $\beta_{t}$ values of up to 40% [6]. These are approximately an order of magnitude higher than those observed in conventional aspect ratio (*A* approx. 3) tokamaks. The choice of A is in principle a continuum. But above a certain aspect ratio A≥ (1.8–2.0), an inboard breeding blanket is necessary to get a sufficient tritium breeding ratio of approximately 1.1–1.2 [7,8]. The STEP design tries to avoid the use of an inboard breeder to reduce complexity. In addition, the small centre column enables a compact build and efficient use of the toroidal field. STs have a high natural elongation $\kappa_{\text{nat}}$ and can reach high normalized beta, $\beta_{N}=\beta_{t}\left(\frac{aB_{t}}{I_{p}}\right)\leq 7$ ( $I_{p}$ is plasma current). For example on NSTX κ≲2.7 and $\beta_{N}≲7$ have been observed simultaneously in the highest performing discharges [9]. This makes it possible to reach high fusion power in a compact design. For a non-inductive (steady state) tokamak with a high self-driven current fraction (bootstrap fraction $f_{\text{BS}}$ ) the fusion power scales like $P_{fus}/V_{pl}\propto \frac{1}{A}{\left(\kappa \beta_{N}B_{t}\right)}^{4}$ [7,10]. However, with respect to the Lawson criteria current STs are far from break-even owing to their small size and extrapolations are large. The energy confinement time observed in present-day ST experiments is broadly in line with scaling laws for conventional aspect ratio tokamaks [11,12], though scaling laws based only on ST data suggest a weaker scaling with $I_{\text{p}}$ and a stronger scaling with $B_{t}$ [13,14] or the dimensionless quantities such as normalized collision frequency $\nu^{⋆}$ [15,16], safety factor q [15] and β [17]. These scaling laws would extrapolate favourably to STEP but need to be treated with care owing to the properties of the turbulent transport in STEP (see §4.a).

While the global plasma properties of an ST are beneficial for a reactor, the compactness of the device, in particular of the centre column, poses some key challenges for the plasma. The restricted space in the centre of the device does not allow for a sufficiently large solenoid to reach the plasma currents required for the flat-top operating point (FTOP). Therefore, non-inductive operation is mandatory for the ST even during the current ramp-up and ramp-down phases. This requires operation at high $f_{\text{BS}}$ and efficient non-inductive current drive (CD) methods to minimize the power requirements for the auxiliary HCD systems. This high Q≲10 , high $f_{BS}≲(0.8-0.9)$ operational point is a highly self-organized nonlinear system that is difficult to control and the control concepts and challenges are discussed in [18] as well as the paper ‘Controlling a new Plasma Regime’ in the same issue [19]. Another plasma challenge is the exhaust of particles and heat through the very thin scrape-off layer (SOL) with a width of only $\lambda_{\text{SOL}}\approx$ 1–2 mm [20–22] (see §3.b) at the required plasma current and the small radius of the strike points. Hence, novel divertor concepts are needed for the plasma exhaust.

There have been several previous studies that take a low toroidal field ST as the basis for a conceptual power plant design, including STPP [23–25], ST pilot plants and FNSF [7,26], ARIES-ST [27] and VECTOR [28,29], or use the option of the high toroidal field in an ST [30], with aspect ratios in the range 1.4≤A≤2 . The plasma parameters for these designs, aiming for very compact solutions, sometimes with resistive coils [24], are often quite extreme, ARIES-ST for example assumes $\beta_{N}=7.4$ with a plasma current of $I_{p}=29\;MA$ in an R=3.2m , A=1.6 device and STPP has $\beta_{N}=8.2$ with $I_{p}=31\;MA$ , R=3.4m and A=1.4 .

In the following sections, we will discuss the basic design philosophy, the major design drivers, the challenges that arise from fully non-inductive operation during the flat-top phase of a pulse, before describing the major gaps in the physics basis and outlining the path to improve confidence in the solutions. While this paper concentrates on the design philosophy, many of the quantitative assessments shown have been carried out for specific scenarios, which are described in more detail in [1,10]. Most commonly a high density (HD) $f_{\text{GW}}=\bar{n}/n_{\text{GW}}\approx 0.95$ ( $n_{\text{GW}}=10^{20}\frac{I_{\text{p}}\text{[MA]}}{\pi a^{2}}\;{\text{m}}^{-3}$ : Greenwald density), $I_{\text{p}}=21\;\text{MA}$ , $P_{\text{HCD}}=P_{\text{EC}}=150\;\text{MW}$ , Q=10 electron cyclotron (EC) resonance heated only point with a radiated power fraction in the core of $f_{\text{rad}}=\frac{P_{\text{heat}}}{P_{\text{rad}}^{\text{core}}}=70\%$ has been used (EC-HD). Figure 1 shows an equilibrium plot and indicates typical parameters for this design point.

## 2. Basic design philosophy

The overall philosophy for the STEP plasma scenario design is to facilitate the key benefits of the ST while aiming to be as conservative as possible with the assumptions. Nevertheless, at the beginning of the project, a wide trade space was explored, and a broad range of scenario families and attributes were assessed for their merits, such as

— Positive triangularity (PT) versus negative triangularity (NT).
— Different HCD schemes and mixes.
— Double null (DN) versus single null (SN) diverted solutions.
— H-mode-like edge versus L-mode or I-mode-like edge.
— The presence of internal transport barriers.

The above characteristics have a somewhat binary character but most of the performance-defining quantities have a continuous spectrum. For these, optimization paths were defined, and specific parameter scans were performed to understand the interaction between the different optimization strategies. The parameters for the viable plasma scenario are also constrained by the choice of HCD system (e.g. $B_{\text{t}}$ and plasma density $n_{\text{e}}$ for RF or microwave-based HCD schemes), plasma control and engineering and cost considerations.

The assessment was done for the FTOPs only based initially on a zero-dimensional plasma description in the system code PROCESS [31]. For promising designs and to understand interdependencies within the plasma better, the one-dimensional transport solver JETTO [32] was used as detailed in [33] and [10] to map and optimize the parameter space. Reliable reduced transport models appropriate to STEP-like conditions do not yet exist (see §4.a). Therefore, JETTO is used in ‘assumption integration’ mode to build a one-dimensional description of a steady-state non-inductive plasma (zero loop voltage) with self-consistent profiles, transport, heating, CD and fusion sources, impurities, radiation and fuelling, with two-dimensional fixed boundary equilibrium. The boundary shape is iterated to be consistent with free-boundary modelling. Normalized beta ( $\beta_{N}$ ) is used as an input parameter, and the gyro-Bohm (gB) transport is adjusted until the target is met. The coefficients of the gB model are set with heat transport dominated by the electron channel, as expected for the hybrid-kinetic ballooning modes and microtearing modes found in STEP conditions (§4.a). Studies using an extended equilibrium code SCENE coupled with linear gyrokinetic calculations provided guidance for beneficial equilibrium quantities in the core [34]. To achieve the required fusion performance, here $P_{\text{fus}}\geq 1.5\;\text{GW}$ for net power production largely determined by the effective thermal cycle efficiency and stationary losses [35] in a compact design, a PT plasma with a DN primary divertor configuration and a narrow transport barrier at the edge (H-mode like) is the most promising candidate. H-mode [36] is ubiquitous in diverted tokamak devices when the ion heat flux through the separatrix exceeds a certain value but often exhibits edge localized modes (ELMs) that require special attention for reactor-relevant devices. There is no predictive theory for the conditions that lead to the formation of an edge transport barrier (H-mode like). In the modelling, the usual Martin *et al*. scaling law [37] is used to initiate transitions between L- and H-mode, but STs may behave differently [38] (see also to §4.d). The power flux through the edge in the FTOP exceeds the Martin scaling law by a factor of 2 or more even with 70% of the power radiated in the core, while the ion heat flux according to a recent scaling law [39] (adjusted for the isotopic mass dependence) would be marginal, but the extrapolation has a very high uncertainty. For the HCD, microwave-based methods that exploit the EC resonance give the overall best performance with respect to wall plug efficiency, design integrability and access to all radii [40]. In an ST these are the EC heating and current drive and the electron Bernstein wave heating (EBW) and electron Bernstein current drive (EBCD).

The pressure of the pedestal forming in H-mode is estimated using a scaling $p_{ped}\propto I_{p}^{0.8}f_{GW}^{0.45}$ derived from the pedestal prediction code EPED1 [41] for STEP-like conditions using the kinetic ballooning mode (KBM) constraint on the pedestal width of $\Delta_{\text{ped}}=0.1\beta_{\text{p,ped}}^{0.5}$ ( $\beta_{\text{p,ped}}$ : poloidal β at the pedestal top) based on the analysis done on the experimental MAST pedestals [42]. It should be noted that on NSTX a different pedestal width scaling of $\Delta_{\text{ped}}=0.43\beta_{\text{p,ped}}^{1.03}$ backed by gyrokinetic modelling is found [43]. The height of the pedestal in the integrated modelling is reduced by approximately 20% from the EPED1 predictions to account for the pedestal being without type-I ELMs and not reaching the peeling-ballooning boundary. The CD profiles use simple scaling with a fixed normalized CD efficiency $\zeta_{\text{CD}}\propto T_{e}/n_{e}$ calibrated against more sophisticated modelling (§3.c) and the heating profile is adjusted to give the desired *q* profile (§3.d) using a genetic algorithm [44]. The fuelling is assumed to be dominated by pellets with a deposition profile around a normalized minor radius ρ (approximately 0.7); edge pellet fuelling, studied experimentally in MAST [45], will be critical in a tokamak power plant because it will provide the dominant particle source. The particle confinement time is assumed to be $\tau_{\text{P}}∼4\;\tau_{\text{E}}$ consistent with the experience on conventional aspect ratio tokamaks. Limited particle transport studies that exist for STs are reviewed in [17]. A first flux-driven transport prediction for a STEP flat-top, using a new reduced model for turbulent transport from hybrid-KBMs, finds that at the transport steady state *τ*_P_/*τ*_E_ = 2.7 [46], i.e. reasonably close to the JETTO assumption. The JET experiments with trace tritium and fuelling source perturbations from neutral beam injection (NBI) and gas puffing [47,48] found $200\;\text{ms}\leq \;\tau_{\text{p}}\leq 800\;\text{ms}$ , with an inverse dependence on β . In this work, it was experimentally inferred that the ion particle diffusivity is lower than the thermal conductivity and that there is an inward pinch velocity. The latter is routinely found in first-principle turbulent transport models. The energy confinement times of the pulses are not uniformly reported in these papers but can be computed from the presented time traces of heating and stored energy (or looked up directly in the JET database) and are approximately $\tau_{\text{E}}∼200\;\text{ms}$ , also typical of JET H modes at these powers. So while these experiments are not with pellets, they provide support for the predictive modelling with quasilinear transport models of STEP [46] and JET [49] which both find particle confinement times are 3–4 times longer than the energy confinement time when a core fuelling source is dominant. Note that the particle confinement time was not reported in [49] but checking these simulations, retrospectively, it is found that the NBI fuelling is dominant in the simulation and $\tau_{\text{p}}\sim 3.8\tau_{\text{E}}$ . While STEP may have quite different transport to JET, these comparable results in modelling give more confidence to use the JET experimental results as motivation for the assumption used on particle confinement. Perturbative experiments with impurity injection also find impurity confinement times longer than energy confinement times [50].

Attempts on MAST experiments to quantify a ‘pellet retention time’ experimentally by a similar methodology [11] found that $\tau_{\text{p}}\geq \tau_{\text{E}}$ only in ELM-free plasmas (which STEP will be). This analysis is complicated by the large perturbation in the overall density profile and transport properties of a pellet in MAST. The individual pellet perturbations should be relatively smaller in STEP. The STEP modelling so far published has assumed a continuous particle source, and the dynamic effects of these perturbations on transport and particle confinement time remain as future work to be analysed in STEP scenarios in conjunction with a first-principle transport model.

If the particle transport assumptions used to design the present STEP scenarios require subsequent updating, it will mostly result in a change in the pellet fuelling demand. This would not be a significant problem unless $\tau_{\text{p}}$ differs by more than an order of magnitude from the assumptions. If $\tau_{\text{p}}$ is too low than the tritium burnup fraction will be too low, causing problems for the fuel cycle to reprocess and reinject enough tritium in the burning steady state. If $\tau_{\text{p}}$ is too high than the helium ash is likely to accumulate, overwhelming the pumping capability and choking the fusion burn.

Argon is needed for the exhaust solution and a content of 0.5% is assumed. Helium ash production is computed self-consistently with fusion reactions giving approximately 9% saturated content. Xenon is assumed to be seeded within the pellets and the content is adjusted to give a core radiation power fraction $f_{\text{rad}}\approx 70\%$ (larger core radiation fractions are assumed to lead to control problems). The first wall material in the SPP will probably be tungsten. For permissible W concentrations in the plasma, the radiation from W can probably be compensated by reducing the Xe concentration. Ideally, the core radiation fraction is minimized, but the plasma exhaust requirement of $P_{\text{sep}}/R∼40\;MW/\text{m}$ sets an upper limit for the power crossing the separatrix $P_{\text{sep}}=P_{\text{heat}}-P_{\text{rad}}^{\text{core}}$ . The JETTO runs are progressed to a steady state with a fully relaxed current profile, producing outputs of plasma current, plasma equilibria, kinetic profiles, safety factor ( q ) profile, CD profiles, $f_{\text{BS}}$ , fusion power and Q [10]. In assumption integration mode, confinement is assumed, and auxiliary power and fusion gain are determined by the steady-state CD power requirements. If the confinement is predicted and not assumed with $\beta_{N}$ as input, then the fusion gain is extremely sensitive to the confinement assumptions but increases non-linearely with CD power at fixed Greenwald fraction (as shown by the simple predictive simulations [1]). As no validated predictive reduced model for the STEP parameters yet exists in these simulations a simplified set-up was used and the sensitivity to certain confinement assumptions was tested, such as artificially doubling the predicted electron or ion transport, or reducing the pedestal pressure, or varying the core radiation fraction. For the FTOP, only the steady state is considered.

The strong scaling of fusion power with κ and $\beta_{N}$ drives the design to a solution with a broad current profile, high elongation and high plasma pressure. The latter is best achieved with high triangularity as the achievable stable pedestal pressure increases strongly with triangularity (see also §4.b) and an H-mode-like edge. The plasma confinement is generally determined by the plasma current [51], which in a non-inductive burning plasma is determined by the fusion power itself and the auxiliary HCD. The integrated modelling shows that the confinement assumption is strongly related to the auxiliary CD efficiency. This is another reason why heating systems with high CD efficiency at high density are very beneficial for fusion power plants (see §3.c).

The scenarios are routinely checked for fixed boundary ideal magnetohydrodynamic (MHD) stability to n = 1–3 modes, and to infinite- n ballooning modes. As discussed in the following text, the low aspect ratio and choice of q -profile is beneficial in giving strong stability to neoclassical tearing modes (NTMs). Also as discussed in the following text, and the companion paper on plasma control, resistive wall modes (RWMs) may be unstable and so RWM control coils are being explored [19].

## 3. Major design drivers

There are various options in designing the FTOP that have a major influence such as NT versus PT configurations, the plasma exhaust requirements, the HCD requirements, the choice of the q-profile and its implication to plasma stability, the effects owing to the toroidal field design or the challenges of requiring a largely non-inductive plasma solution. These are briefly discussed in the following sections.

### (a) NT versus PT

Plasmas with NT were considered as an option for STEP. Such plasmas have been found to have some attractive confinement properties in conventional aspect ratio tokamaks (refer e.g. to [52]) and are naturally ELM-free. They are also beneficial for the plasma exhaust as the target area is increased. However, the MHD stability properties of NT plasmas are much worse than those of PT plasmas. In the PT case, the design relies on access to second ballooning mode stability (see the following sections) but for NT, this is not possible [53], and NT also leads to a greater restriction on the core pressure to avoid strong KBM instability [53]. Furthermore, internal low n instabilities are found to dominate over external kink modes (which result in RWM); this is similar to results found in conventional aspect ratio tokamaks [54]. Consequently $\beta_{N}$ is optimized by increasing the magnetic shear in the core, with the central safety factor $q\left(0\right)\sim 1$ , but is still limited to $\beta_{N}\sim 3$ . The lower q(0) leads to higher internal inductance in optimized NT plasmas compared with those of PTs, and a limit on the achievable plasma elongation that is approximately 30% lower. In summary, the key ST advantages of high κ and $\beta_{N}$ cannot be realized with NT, and for this reason, it was decided that STEP plasmas should have PT.

### (b) Managing the exhaust

The burning plasma phase presents a clear exhaust issue for STEP, with a total heating power of $P_{\text{heat}}\approx \frac{P_{\text{fus}}}{5}+P_{\text{HCD}}∼500\;\text{MW}$ and considerable He generation, occurring over hours of steady-state operation per day. However, unlike conventional reactor designs, such as DEMO, that use a solenoid to drive plasma current, the non-inductive ramp-up phase also presents a significant exhaust challenge in STEP, comparable to the burning phase, owing to the need for high auxiliary power injection of the order of $P_{\text{HCD}}≲200\;\text{MW}$ for external CD derived from JETTO calculations [1].

**Figure 2.**
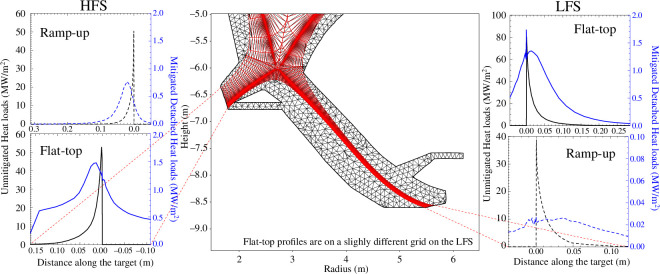
SOLPS-ITER grid (middle) and typical unmitigated (black) and mitigated (blue) heat load profiles in the flat-top (solid) and current ramp-up (dashed) at $I_{\text{p}}=10\text{MA}$ .

To distribute the heat over a large area, impurity seeding is used; Xe is foreseen as the main core radiator and Ar is used in the edge and SOL. Up to 70% of the loss power is anticipated to be radiated before crossing the separatrix, with the radiation increased above the inherent background continuum radiation (i.e. bremsstrahlung and synchrotron) via injection of Xe-doped pellets, leaving approximately 150 MW of remaining power to be exhausted. This remaining power is transported by particles following open field lines in the SOL plasma surrounding the core plasma towards both an inboard and outboard high heat flux handling component known as the divertor. The area over which the power is spread scales with the divertor strike-point radius, the integral power decay length, and the ratio of the poloidal to the total magnetic field. Therefore, while a higher fraction of power is inherently directed to the outboard divertor, the small inner divertor strike-point radius in STEP, characteristic of a compact ST, results in significantly higher heat and particle fluxes at the inboard divertor. The design of the divertor can either follow a conventional SN design, following the foreseen geometry in ITER and DEMO, or an alternative divertor configuration. Alternative divertor configurations typically assessed in current machines (e.g. MAST-U [55]) include a DN configuration, extended divertor strike-point radii, and the inclusion of an additional X-point near the targets. Figure 2 shows the current design of the divertor in the modelling.

An SN configuration uses a single set of inboard and outboard divertors located either at the bottom or top of the machine, while a DN configuration uses two sets of inboard and outboard divertors located at the top and bottom of the machine to increase the area over which the power is spread. It is acknowledged that achieving a connected DN (CDN) configuration with both X-points on the same flux surface (separatrix) is impossible. Indeed, a dynamic DN oscillating on the vertical control time scales around a CDN is the standard operating mode in DN tokamaks such as MAST(-U). Still, the advantage of the DN configuration is only lost when the separation between the two separatrices at the outboard midplane exceeds $\Delta r_{\text{sep}}^{\text{out}}>2\;\lambda_{\text{SOL}}$ [56,57] and SOLPS-ITER studies have been performed on STEP to quantify accuracy required for vertical control [58]. Furthermore, the oscillations will result in a time-average power loading of the different divertors. It should be noted that to achieve the required accuracy in $\Delta r_{\text{sep}}^{\text{out}}$ the vertical position needs to be controlled to $\Delta z≲\left(8-10\right)\lambda_{\text{SOL}}=O\left(1\;\text{cm}\right)$ . Nevertheless, the divertors are engineered to transiently withstand SN heat and particle loads. Compared with DN, the SN configuration provides clear benefits: there is easier maintenance access to the machine end opposite to the divertor, there is half the number of components to fail in the divertor, and it reduces the complexity of the vertical position control system as less accuracy is required. However, for perspective, if the SN conventional divertor geometry were scaled into STEP, then the parallel particle flux at the outboard divertor target would be approximately double that expected in DEMO and triple at the inboard divertor, assuming equivalent upstream plasma conditions. Furthermore, owing to the smaller machine size of SPP, there would be major spatial integration challenges with incorporating, around a single divertor, the 24 cryogenic pumps [59]. To exhaust the He in the current design, an effective pumping speed of $S_{\text{pump}}∼30\;{\text{m}}^{3}/\text{s}$ is required in the SOLPS-ITER modelling. The study also showed that the requirements during the non-inductive ramp-up would lead to a very large device negating the advantage of the ST. Here, also a dynamic DN configuration is assumed, but the control accuracy requirements become less demanding as the power flux width increases with decreasing plasma current [20]. Therefore, in summary, the SN configuration was ruled out for STEP and an up–down symmetric DN configuration was adopted. However, maintenance access and accurate vertical position control remain issues with various solutions still under consideration.

To achieve a SOL plasma solution in STEP compatible with material limits the divertor plasma needs to be detached from the targets. Divertor plasmas are labelled as ‘detached’ when large pressure gradients are observed parallel to the magnetic field, resulting in low plasma power and ion fluxes to the material surfaces [60], and typically coinciding with low electron temperatures ( <5eV ) at the divertor target. Extending the outer divertor strike-point radius, with tight baffling of the divertor region, provides the benefit of widening the detachment window, reducing the demand on the Ar seeding by approximately 30%, and increasing the level of power transients that can be buffered. An equivalent extension of the inboard divertor radius is not possible, and therefore an additional X-point was introduced near the inner divertor target, but outside of the wall boundary (i.e. approaching an X-divertor). This configuration results in flared flux surfaces at the target, providing a more even distribution of recycled neutrals and thus facilitating detachment across the entire divertor target [61]. However, since only the outboard divertors are pumped, the increased number of neutrals recycled into the SOL in this configuration results in higher numbers of impurities (e.g. He) travelling upstream into the core plasma, in comparison to the conventional divertor geometry. An ITER-like dome structure [62], providing a passage between the inner and outer divertors without a direct line-of-sight to the core plasma, has been implemented to maximize the number of recycled particles reaching the outboard pumps. A range of predictions for the power width have been examined, incorporating empirical scalings and theoretical models such as the Heuristic Drift (HD) model by Goldston [21], alongside standard [20] and ST aspect-ratio [22] scaling laws. The empirical predictions for STEP, based on standard aspect ratios, yield the lowest values ( $\lambda_{\text{SOL}}$ approx. 0.5 mm). However, the HD model predicts larger widths ( $\lambda_{\text{SOL}}$ approx. 2 mm), and empirical predictions from MAST indicate even larger widths ( $\lambda_{\text{SOL}}$ approx. 4 mm). Stability analysis indicates that the lowest empirical predictions for the width $\left(\lambda_{\text{SOL}}≲0.5\;\text{mm}\right)$ are unlikely, considering MHD stability scaling laws: narrow SOL widths would be ideal MHD unstable at moderate separatrix densities $n_{\text{sep}}\sim \left(0.2-0.3\right)n_{\text{GW}}$ . At a separatrix density of $n_{\text{sep}}\approx 0.3n_{\text{GW}}$ , stability analysis suggests a KBM marginal power width of 1.5 mm, with an ideal MHD limit at approxomately 1.1 mm. Thus, we have adopted an overall range of $1\;\text{mm}\;<\;\lambda_{\text{SOL}}\;<\;2\;\text{mm}$ as the basis for designing STEP, aiming to remain conservative. Our simulation transport coefficients are configured to ensure power widths within this range. Despite the advanced divertor design, the concentration of Ar required to dissipate the remaining power directed to the divertors still remains high, ranging between 2 and 4% throughout the current ramp-up and flat-top, depending on the assumption of radial transport coefficients set as input in SOLPS-ITER simulations [58]. It is estimated that the core Ar concentration must be below 0.5% to avoid significant dilution of fuel. Current experiments typically measure a factor-two decrease in Ar concentration between the SOL and core [63,64]. Modelling of the STEP edge for the FTOP with NEO suggests that the strong temperature gradient in the plasma edge in STEP will drive an outward neoclassical flux as seen experimentally and predicted for ITER [65,66], thus screening the core of Ar beyond the values measured in current machines; however, the extent of this screening is so far unknown. Increasing the divertor pressure could be considered to reduce the demand on the Ar puffing [67,68]; however, this may not be compatible with the ramp-up phase, which would require low divertor pressure (hence low plasma density). In summary, the chosen divertor design and exhaust plasma solution in STEP presents a potentially viable integrated scenario with sufficient fusion power gain, albeit with notable challenges, such as high Ar concentration, maintenance and vertical control, that need further investigation.

### (c) Constraints owing to the HCD scheme

STEP uses (exclusively) microwaves for the HCD actuators. For the ST, this means either an electron cyclotron current drive (ECCD) or an electron Bernstein wave current drive (EBCD). This decision resulted from a detailed assessment of current drive (CD) efficiency, electrical efficiency, functional suitability, technological readiness, cost, reliability, availability, maintainability and inspectability [69]. The study showed that while the CD efficiency for neutral beam injection (NBI) is with approximately 40A/W for 0<ρ<0.5 and 0–40 A/W for 0.5<ρ<1 is similar to ECCD ( 40A/W , 30A/W , respectively); the wall plug efficiency is much lower and the technical implications are more difficult. The helicon wave would offer relatively high CD and wall plug efficiency can only access ρ>0.65 and does not reach the performance projected for EBCD. Owing to its maturity and felxibility ECCD is the primary CD actuator, while EBCD is maintained in the design process primarily because of predictions that it would deliver a much higher CD efficiency. It would also allow operation at higher density and thus higher fusion power [40,70]. It should be noted that ECCD and EBCD have not been tested on the largest high-performing STs yet and are mainly studied in small devices. However, on MAST-U a ≳1.4MW EBCD system is planned [40,71,72] to validate the EBW coupling and CD physics in an ST and ST40 will soon be equipped with an approximately 2MW power ECCD system [73] for physics validation.

**Figure 3.**
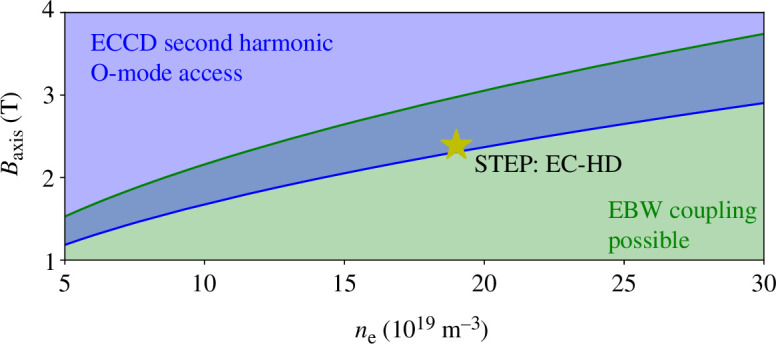
Limitations for ECCD and EBCD access. The blue (dark grey) region indicates the area of parameter space where second harmonic O-mode access is possible, while the green region (light grey) indicates where LFS EBW coupling can occur. The star indicates where the EC-HD FTOP lies in this parameter space and field and density for this point are calculated at the magnetic axis. In a narrow band in density and field the regions overlap.

The use of ECCD plays an important role in the selection of the magnetic field and density combination in the STEP prototype. It was initially assumed that the second harmonic ordinary (O)-mode would be used, requiring as a minimum the second harmonic to be above the O-mode cut-off at the magnetic axis. For EBCD, a low-field side (LFS) ordinary-extraordinary-Bernstein (O-X-B) mode coupling would be used [74], requiring the fundamental to be lower than the O-mode cut-off. As a design guide, for EBCD access, we have used the more stringent condition that the left-hand X-mode cut-off is above the fundamental at the magnetic axis. This has the effect of ensuring that the coupling layer is towards the outer layers of the plasma, which support higher density gradients and where higher coupling efficiency can be expected. Figure 3 shows the region of overlap between these constraints and where the EC-HD FTOP [1,10] sits. From an ECCD perspective, second harmonic O-mode access to the core is guaranteed by this approach and fundamental O-mode will also be accessible from the high-field side (HFS) of the device.

The CD efficiency in units of A/W, $\eta_{\text{CD}}$ , can be usefully normalized to remove variations with radius, temperature and density [75]:


(3.1) $$\zeta_{\text{CD}}=3.27\frac{R_{m}n_{19}}{T_{keV}}\eta_{\text{CD}},$$

where $R_{m}$ is the major radius of the geometric centre of the outermost flux surface in *m*, $n_{19}$ is local electron density in units of 10^19^ m^−3^ and $T_{\text{keV}}$ is local electron temperature in keV. For the EC case, the assumption that $\zeta_{\text{CD}}≃0.2$ , and is approximately constant across the plasma, is used to quickly develop an operating point without the need for extensive beam-tracing analysis. The validity of the assumption is then checked with extensive scans of the frequency, position and launch angle of EC launchers using the GRAY beam-tracing code [76]. The results of this analysis can then be used to recalibrate $\zeta_{\text{CD}}$ if necessary, and also find the optimal launch configuration for that scenario, which then also guides the EC engineering design [71]. For the EC-HD FTOP, it is found that the auxiliary current profile can be matched most efficiently by using a range of frequencies, with four to five frequencies being recommended. In addition, at least one frequency above 170 GHz is required for the target efficiency. EBCD uses frequencies in the range of 90–100 GHz, and relativistic ray tracing shows that it can only be used to access the core at low parallel refractive index [77], which leads to low CD efficiency. Fokker–Planck simulations using the bounce-averaged, relativistic code CQL3D [78] show the off-axis CD (at ρ>∼0.6 ) for EBCD is dominated by the Ohkawa mechanism on the LFS of the plasma and exceeds the ECCD efficiency by at least a factor of two [70].

Overall, the HCD system needs to have sufficient redundancy and margin for both heating schemes (ECCD only and EBCD+ECCD). In a non-inductive burning plasma scenario, the fusion power at constant Greenwald fraction has a strong nonlinear increase with the HCD power [1]. Oversizing the HCD system mitigates the risk of an underperforming plasma. Furthermore, to achieve relevant plasma currents during the no (H) or ‘low’ activation (D) phases that are required for plasma commissioning the missing fusion power and bootstrap current have to be replaced by the HCD system. Therefore, the HCD system is being sized to provide 300 MW of injected power. So far only a few scenario simulations for the commissioning phase have been done indicating that such power levels should be adequate.

### (d) Choice of safety factor profile

The safety factor profile, q , is chosen to be monotonic with $q_{\text{min}}\geq 2.3$ , and maximized shear on rational surfaces. Additional constraints are discussed in [79]. The choice of $q_{\text{min}}$ is consistent with high $f_{\text{BS}}$ and with low internal inductance $0.25\;<\;l_{i}\left(3\right)\;<\;0.3$ needed to allow high κ plasmas. It also avoids the low-order resonance at q=2 , where potentially disruptive NTMs may occur. Furthermore, elevated $q_{\text{min}}$ is consistent with direct access to second ballooning mode stability [80]. Studies to optimize the q -profile are yet to be conducted, and it may be the case that higher $q_{\text{min}}$ values than those used so far are found to be optimal, as shown in [81]. Nevertheless, the q -profiles of the equilibria used so far provide a good basis for STEP FTOPs: they are stable to internal MHD instabilities and NTMs with n=1 and 2. Modelling of NTMs, validated against experimental results from several devices including the MAST ST [82], shows strong curvature stabilization at a low aspect ratio [83], which suppresses the excitation of NTMs with m=3 , n=1 and m=5 , n=2 . Since the equilibria tend to have low shear in the core, it is found that $q_{\text{min}}$ above approximately 2.2 is necessary to prevent infernal mode [84] from being either unstable or close to instability and coupling to RWMs.

### (e) Constraints for the toroidal field design

It is envisaged at present that STEP will have picture-frame toroidal field (TF) coils with vertical outer limbs [85]. In such cases, the toroidal ripple in the equilibrium magnetic field arising from the use of a finite number of coils N is a function of major radius only inside the plasma, and the perturbations to the field components in right-handed cylindrical coordinates (R,φ,Z) can be well-approximated by the expressions [86]


(3.2) $${\tilde{B}}_{R}=\frac{B_{0}R_{0}}{R}{\left(\frac{R}{R_{\text{coil}}}\right)}^{N}\sin N\varphi ,\;\;\;\;\;\;\;\;\;\;\;{\tilde{B}}_{\varphi }=\frac{B_{0}R_{0}}{R}{\left(\frac{R}{R_{\text{coil}}}\right)}^{N}\cos N\varphi ,\;\;\;\;\;\;\;\;\;{\tilde{B}}_{Z}=0,$$

where $R_{\text{coil}}$ is the outer limb coil radius and $B_{0}$ is the field at the magnetic axis, $R=R_{0}$ . Any such perturbations violate the conservation of toroidal canonical momentum and thereby degrade fusion α-particle confinement. Any non-axisymmetric ferromagnetic structures in the tokamak assembly will also modify the magnetic field; but are not considered here.

**Figure 4.**
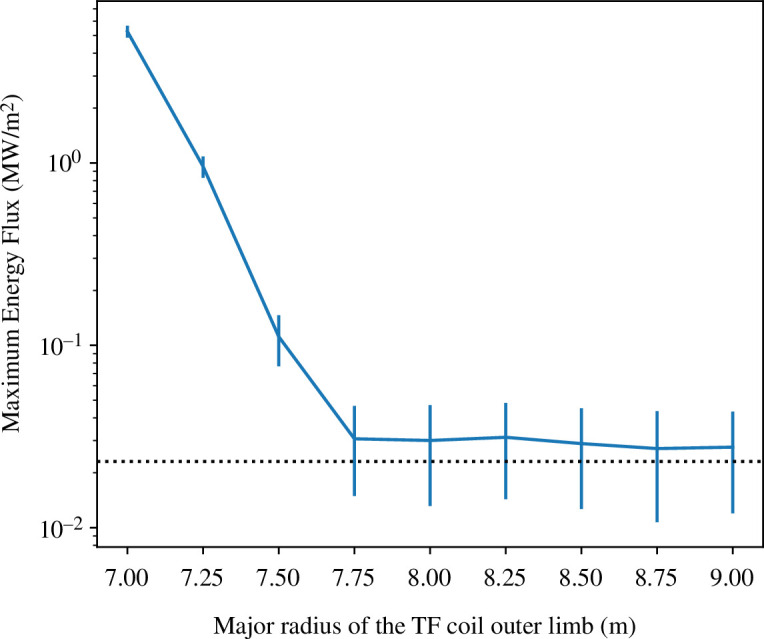
Maximum power loading on the first wall owing to prompt and TF ripple-induced α -particle losses versus TF coil outer radius for the case of 16 coils.

The choice of N is determined largely by engineering constraints, in particular, ease of maintenance and port access; the baseline value of this parameter is 16. It is typically assumed that fast ion losses are likely to be acceptable if the ripple amplitude $\delta =\tilde{B}/B_{0}$ does not exceed a value of approximately 1% anywhere in the plasma. For the field perturbation given by equation (3.2), the maximum occurs at the outer midplane plasma edge, which in currently used STEP FTOPs is R≃5.6m . It can be inferred from equation (3.2) that a minimum coil radius satisfying this condition is approximately 7.5m . This is a useful first estimate, but a reliably safe value of $R_{\text{coil}}$ can only be calculated by simulating α-particles for about a collisional slowing-down time from birth in a realistic equilibrium field with the ripple perturbation superimposed, and using a model for the first wall so that precise loss criteria can be defined, and α-particle power loading maps can be generated. For this purpose, we have used the GPU-based LOCUST code, which can track a sufficiently large number of markers to provide accurate estimates of the maximum power loading [87]. Figure 4 shows the maximum α-particle power loading versus $R_{\text{coil}}$ for N=16 . This illustrates the high sensitivity of the ripple-induced losses to the perturbation amplitude and confirms that $R_{\text{coil}}$ for this number of coils should be approximately 7.5 m or more; the peak loading in this case occurs on the tungsten armour on the LFS of the main chamber wall where the maximum tolerable load owing to all non-neutronic sources (thermal plasma, high energy α-particles and electromagnetic (EM) radiation) is approximately 1–2 MWm^−2^.

These results depend on the exact values of $B_{0}$ (field on the magnetic axis) and $I_{\text{p}}$ (respectively, 2.5 T and 20.5 MA for the FTOP used here), since these determine the α-particle orbit width. We have found that adding limiters to the model used for the first wall has no effect on prompt α-particle losses. The TF ripple-induced losses occur predominantly in a region of the main chamber where no limiters are planned, and therefore, it is expected that the locations of these structures will not affect α-particle-induced wall loads. It should be noted that α-particles can also in principle be lost owing to both self-driven instabilities (toroidal Alfvén eigenmodes (TAEs) [88], for example) and unstable modes of the bulk plasma (such as NTMs and RWMs). Analysis so far indicates that TAEs in the STEP FTOPs have a very strong drive from the α -particles but their Landau damping by bulk ions is, according to HALO calculations, even stronger [88]. As the net damping rate is a balance between two very large numbers, there is some uncertainty on the stability of TAEs in this regime and more validation in high β ST plasmas with strong TAE drive is needed. The effect of controlled RWMs on α-particle confinement is likely to be negligible [88]. The effects of other instabilities will be considered in future work.

Fusion α-particle production will be negligible during ramp-up since the plasma density will be very low during this phase to facilitate ECCD until the full plasma current is reached. A transition to pure deuterium fuelling is envisaged at the start of ramp-down, with a consequent drop in α-particle production but it is assumed that this will take place over a timescale of the order of 100 s while the current will drop continuously during this period; α-particle losses in this phase, which will be determined by the competing effects of a falling birth rate and wider orbits arising from reduced current, have yet to be modelled.

### (f) Challenges for the non-inductive scenario

The space in the centre of an ST power plant is very precious and implementing a solenoid that allows the generation of the full plasma current is impossible with the current technologies. While a design without a solenoid would be preferable, a small solenoid can mitigate the considerable risk of not being able to form a plasma. Non-solenoidal start-up schemes have been studied for STs [89] experimentally and is a very active field of research, but the theoretical framework to allow extrapolation to STEP is missing. A small solenoid, $\Delta \psi_{\text{CS}}∼9\;Vs$ , is foreseen as a proven start-up technique that can be accurately modelled with the DYON code [90] and for the inductive ramp-up to a low current full bore-diverted target plasma for the non-inductive ramp-up [91,92]. In the break down and burn-through phase, oxygen concentrations of 2 and 3% with and without EC assistance at 1mPa prefill pressure still give a successful plasma formation and plasma ramp-up to low currents. The influx of W in the limiter phase may pose a challenge as the amount of allowable EC heating will be restricted by the heat flux capability of the inner limiter.

A key challenge for the non-inductive current ramp-up is the avoidance of a current hole on the axis. As the resistivity is a strong function of $T_{\text{e}}$ , it is likely to have a broader heat and current deposition profile than the conductivity profile. The auxiliary CD will cause an electric field that tries to counter the CD (back-EMF) owing to Faraday’s law. This will drive a counter-current in the plasma with a different profile to the driven current leading to a current hole. To avoid this current hole owing to the interaction between Faraday’s law and the slow current diffusion in a hot plasma, the increase in the plasma current by increasing the auxiliary power has to be done gradually from the centre on the current diffusion time scale. This can be achieved by growing the plasma boundary with a fixed j(ψ) or by broadening the profile using the flexibility of the HCD system [91]. The latter is more advantageous with respect to divertor performance and vertical stability. The overall power of the HCD system needs to be kept as low as possible to keep the heat exhaust in the divertor manageable. The CD efficiency $\eta_{\text{CD}}\propto T_{\text{e}}/n_{\text{e}}$ favours, on the one hand, a hot low-density plasma. On the other hand, the divertor constraint requires a certain minimum density; also the fusion power increases as $P_{\text{fus}}\propto n^{2}$ favouring a high-density FTOP. These contradicting requirements have to be balanced during the ramp-up while additionally maintaining MHD stable current profiles. To achieve net electricity $P_{\text{fus}}\gg P_{\text{HCD}}$ , the overall current must be dominated by the pressure-driven bootstrap current [93]. At fixed heating power, the interplay between density, CD efficiency and bootstrap current leads to a minimum of $I_{\text{p}}$ as function of density [10] which must be overcome to reach the final high-density FTOP. This leads to a ramp-up scenario where first the full current is generated on a long-time scale $O\left(h\right)$ at the lowest possible density and then at full current, the plasma density is increased within 10–100 s to reach the fusion conditions [91]. In small devices with high electron heating, a clamping of the ion temperature has been observed [94] which poses a certain risk to the ramp-up scenario depending on the underlying turbulence. At full current for STEP, the beneficial ratio of energy exchange time to confinement time should make it possible to reach $T_{\text{i}}∼T_{\text{e}}\approx 20\;\text{keV}$ , needed for efficient fusion. To avoid the current hole in the flat-top phase, a small amount of central HCD is also required. The current ramp-down faces different issues, namely, the avoidance of the radiative collapse and maintaining a low $l_{\text{i}}$ . To speed up the ramp-down and to reduce the stored energy in the plasma, it is advantageous to maintain a high density and to reduce the fusion power at high plasma density by changing the fuel from DT to D. In addition, the transition from H-mode back to L-mode needs to be delayed as long as possible to have the corresponding β and $l_{\text{i}}$ change at the lowest possible current.

The toroidal magnetic field on-axis is set by the EC resonance conditions, and thus the minimum ST device size is set by the engineering design of the centre post. Integrated modelling studies for this minimum device size show that, to attain adequate fusion power (approx. $\beta_{N}^{4}$ at fixed $f_{\text{BS}}$ ), $\beta_{N}$ generally needs to be above the no-wall limit for the n=1 external kink mode. Operation in the domain between the no-wall ( $\beta_{N}^{\text{no-wall}}$ ) and with-wall ( $\beta_{N}^{\text{with-wall}}$ ) n=1 kink mode limits means that the RWM is potentially unstable. The exploration of RWM stability for some possible STEP scenarios is discussed in [95]. RWM stability is parameterized by a quantity $C_{\beta }$ defined as


$$C_{\beta }=\frac{\beta_{N}-\beta_{N}^{\text{no-wall}}}{\beta_{N}^{\text{with-wall}}-\beta_{N}^{\text{no-wall}}}.$$

**Figure 5.**
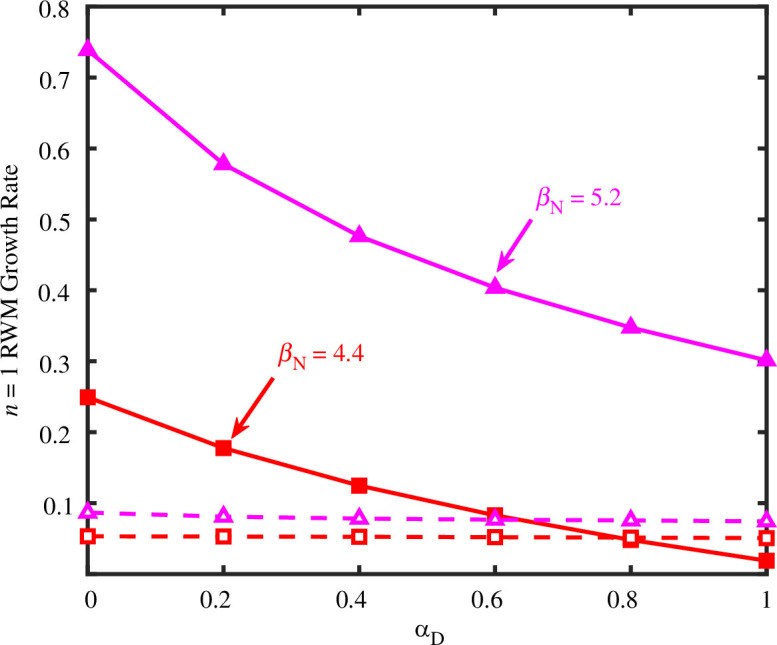
*n* = 1 RWM growth rate (solid, normalized using the wall resistive diffusion time) versus a parameter $\alpha_{\text{D}}$ that controls the degree to which kinetic terms are applied: $\alpha_{\text{D}}$ = 0 is the fluid limit and $\alpha_{\text{D}}$ = 1 is the fully kinetic limit. The red (squares) and pink (triangles) curves are, respectively, for equilibria with $C_{\beta }=0.14$ and $C_{\beta }=0.6$ . The dashed lines show the mode frequency normalised by the wall resistive diffusion time on the same axis.

For $0<\;C_{\beta }<1$ , the RWM is potentially unstable while for $C_{\beta }>1$ , it is ideally unstable (and thus uncontrollable). In the former case, stability can be affected by the presence of toroidal rotation. In the fluid limit, rotation stabilization primarily occurs owing to ion sound-wave damping and coupling to the Alfvén continuum. At low $C_{\beta }$ values, toroidal fluid rotation can completely stabilize the n=1 RWM but, at values above about 0.5, the fluid rotation becomes ineffective in stabilizing this mode [95]. Moreover, since STEP has no external momentum input from the proposed microwave-based schemes, bulk plasma rotation will be low and likely to correspond to Alfvénic Mach numbers that are below the values (typically a few per cent) needed to attain stabilization at low $C_{\beta }$ . The effect of kinetic resonances on stabilizing the n=1 RWM has also been examined. Owing to the low plasma rotation, the dominant kinetic resonance is found to be with the precessional drift of the thermal ions [95]. The strength of this resonance damping on the RWM depends on the intrinsic plasma rotation, which is poorly known. Also, at higher $C_{\beta }$ , the effect of the resonance becomes weak, an example of which is shown in figure 5.

Given the uncertainties of intrinsic stabilization of the RWM by toroidal rotation (see §4.a) or kinetic effects, STEP is designed to have active RWM control. For some candidate STEP FTOPs, it is found that the n=2 RWM can also be weakly unstable. For this reason, the RWM control system is being designed to stabilize both n=1 and n=2 RWMs. The topic of active RWM control in STEP is discussed in the ‘Controlling a new Plasma Regime’ paper, which is part of this journal issue [19].

## 4. Major gaps

### (a) Plasma transport

In combination with the available sources of heat and particles, turbulent transport will determine the equilibrium profile evolution and will be a key driver in dictating the fusion power that can be achieved in STEP. Reduced physics-based models of core transport are essential for integrated scenario modelling, and these are very highly developed for electrostatic turbulence, where extensive gyrokinetic simulations and experimental data are available to guide model development and validation. For example, the construction of TGLF (trapped-gyro-Landau-fluid), one of the most advanced physics-based reduced models of core transport available [96], was closely guided by high-fidelity local gyrokinetic simulations of electrostatic turbulence. STEP is a high *β* ST, for this reason, the turbulence in its core is expected to be strongly electromagnetic (EM), and thus very different in character from the electrostatic turbulence that dominates in the lower *β* regime of conventional tokamaks and has been extensively explored in these devices. Limited studies of EM turbulence using local gyrokinetics have been carried out. These include simulations of EM turbulence in large aspect ratio model equilibria [97], and of micro-tearing mode (MTM) turbulence in the core of the STs NSTX [98] and MAST [99,100] and at the edge of ASDEX-Upgrade [101]. However, large remaining uncertainties in the transport associated with EM core turbulence in STEP mean that plasma confinement is one of the biggest risks to the project.

The transport database for STs is dominated by MAST and NSTX, which have produced plasmas that are useful for confinement studies [17], albeit in non-burning plasma conditions very different from those in the STEP flat-top. In contrast to STEP, NBI typically dominates the heating in MAST and NSTX, giving higher ion heating fractions and stronger toroidal rotation, and the plasmas are at lower β and higher collisionality, $\nu_{*}$ . A strong inverse scaling of energy confinement time with $\nu_{*}$ (stronger than that found at conventional aspect ratio) has been reported independently from NSTX [16] and MAST [15], this is favourable for performance in fusion power plants based on the ST, provided, of course, that the scaling extrapolates. In [16], it is noted that improving confinement at lower $\nu_{⋆}$ in NSTX was largely achieved through a reduction in electron heat transport, and that at low $\nu_{⋆}$ hybrid TEM/KBMs become unstable in the outer plasma where ion thermal transport starts to exceed the neoclassical level.

The highest fidelity model for core turbulence in tokamaks is local gyrokinetics, and this has been exploited, initially neglecting fusion α-particles, to advance our understanding of EM core turbulence in the EC-HD concept design [102,103]. Linear local gyrokinetic simulations close to mid-radius reveal microinstabilities arising at ion scales in the binormal (orthogonal to both the equilibrium magnetic field and flux surface) wavenumber, with dominant hybrid-KBMs and sub-dominantly unstable MTMs [102,104]. Neglecting equilibrium flow shear, nonlinear local simulations find that the hybrid-KBM turbulence robustly runs away to large heat and particle fluxes that exceed the available sources by orders of magnitude, with heat transport that is dominated by magnetic flutter in the electron channel at the lowest binormal wavenumbers [100]. At the finite dimensionless ion Larmor radius $\rho_{⋆}=\rho_{i}/a$ of STEP, in the runaway state, the radial correlation lengths of turbulent structures are in fact too large to be well described by the local equilibrium model. With sufficient perpendicular equilibrium flow shear, transport and turbulence radial correlation lengths are significantly reduced. It should be noted, however, that flow shear is expected to be modest in STEP as there is no momentum source from NBI. Nevertheless, including even a diamagnetic level of flow shear lowers the heat flux closer to the total heat source. We caution that diamagnetic flows strictly require higher order theory in $\rho_{⋆}$ to be included self-consistently. Giacomin *et al*. [103] also report that locally increasing the normalized pressure gradient ( $\beta^{\prime }$ ) reduces the turbulence to a regime where the associated heat transport reaches a better balance with the sources. This is probably to be related to stabilizing effects of more favourable trapped electron precessional drifts [105], and/or to improved stability to ideal n⟶∞ ballooning modes [100]. As this stabilizing effect is reduced at lower pressure gradient, the corollary is that the turbulent fluxes reduce only weakly as the pressure gradient falls, which could challenge access to a burning plasma state if this transport cannot be mitigated. A first flux-driven transport prediction for a STEP flat-top, using a new reduced model for turbulent transport from hybrid-KBMs (and other simplifying assumptions), finds a transport steady state with a fusion power comparable to that assumed in JETTO but does not demonstrate how this can be accessed [46].

A key question affecting transport and stability is the amount of rotation and velocity shear on the flux surface perpendicular to the TF. The intrinsic rotation is a higher-order correction to GK which to our knowledge has only ever been addressed for electrostatic turbulence in large aspect ratio plasmas and still needs theory development for high β EM turbulence. Studies on plasma rotation in STs are usually done in plasmas with high torque input [106–108]. Here, Prandtl numbers of $0.2\leq \frac{\chi_{\phi }}{\chi_{i}}\leq 1$ ( $\chi_{\phi ,i}$ : momentum and ion diffusivity, respectively) are found consistent with values in large aspect ratio tokamaks. Alpha-particle losses arising from three-dimensional field perturbations, such as those owing to TF ripple and resonant magnetic perturbations (RMP; see §4.b), induce an inward radial return current that crosses on the poloidal magnetic field to produce a counter-current toroidal torque on the bulk ions. The effects of this torque on plasma rotation have been modelled using JETTO, assuming Bohm/gB ion energy and momentum transport, and Prandtl numbers of 0.5–1.0. Counter-current rotation rates of up to around 20 km s^−1^ (somewhat less than 1% of the core plasma Alfvén speed) have been estimated in this way. Still momentum transport in high β STs with low torque input remains a key challenge for future research.

### (b) Operating without ELMs

The intermittent instabilities in the plasma edge (ELMs) [109] deposit heat on the divertor and can cause significant damage to the plasma-facing components. In STEP, projections based on scalings from current devices indicate that the heat loads from large ELMs would stay below the melting limit of tungsten but could lead to cracking and erosion of the divertor plates. It is intended that STEP will operate in a regime that has either no ELMs or ELMs that are small enough that damage to the divertor is avoided. Such potential operating regimes include quiescent H-mode (QHM) [110–112], quasi-continuous exhaust (QCE) [113–115] and radiative X-point [116,117]. While these regimes have been observed in current conventional aspect ratio tokamaks, a theoretical understanding of why the ELMs are avoided and how much the confinement is degraded compared with ELMy plasmas is still lacking. Furthermore, their applicability to an ST in DN configuration has not been demonstrated. Nevertheless, the use of Li for wall conditioning led to an ELM-free regime in NSTX [118,119] and recently an ELM-free regime has been observed on MAST-U [120]. Modelling to gain an understanding of these regimes and the ability to extrapolate them to STEP is needed, and in particular, nonlinear MHD modelling of experimental no/small ELM regimes in an ST (as done for conventional tokamaks in QCE [121] and QHM [122] regimes) is required. On the experimental side, the MAST-U tokamak offers a testbed to demonstrate their applicability to STs.

In addition to intrinsically no/small ELM regimes, STEP is being designed to have two sets of coils with coils both above and below the midplane that will produce resonant magnetic perturbations (RMPs) to suppress the ELMs. A preliminary design of the considered three-dimensional perturbation coils for RWM control, ELM control and error field control can be found in [18] and in the ‘Controlling a new Plasma Regime’ paper, which is part of this journal issue [19]. This method has been demonstrated in numerous tokamaks and is planned to be used in ITER. In STEP, it is intended as a backup system in case the no/small ELM operating regimes fail to deliver sufficient ELM suppression. They may also be used under transient conditions such as ramp-up and ramp-down to ensure ELM-free operation. RMP modelling, using a necessary condition for the amplitude of the perturbation that has been found in current devices, projects that a relatively modest current (compared with the system used in ITER) is required in STEP to reach the ELM mitigation conditions [123]. However, it must be noted that full ELM suppression has not been demonstrated either in STs or in DN configuration (the selected plasma configuration for STEP), which makes the above prediction very uncertain. The calculated optimal phase difference for ELM suppression between the currents in upper and lower coils is also not optimal in terms of fusion α-particle confinement [88].

**Figure 6.**
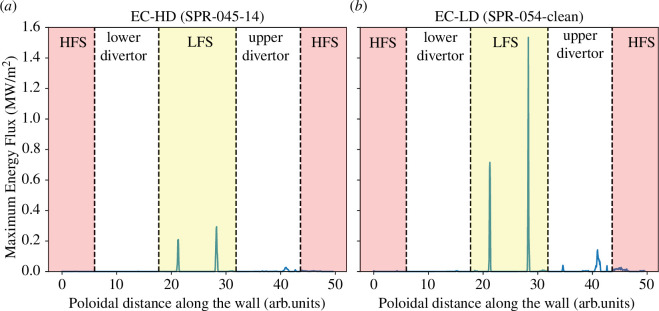
Poloidal distribution of maximum power loading (indicated by the blue line) owing to prompt and TF ripple-induced α -particle losses when $N_{\text{coil}}=16$ and $R_{\text{coil}}=7.4\text{m}$ for (*a*) the EC-HD FTOP and (*b*) a lower-density EC FTOP with a hotter pedestal. The *x*-axis in the plot spans the entire poloidal cross-section of the first wall and divertor.

Losses of fusion α-particles need to be considered in pedestal modelling. The stiffness of energy transport typically found in tokamak plasmas means that high-temperature and density pedestals are highly desirable in terms of optimizing fusion power, but they can also lead to significant α-particle production at the pedestal top. Figure 6 shows the poloidal distribution of power loading owing to axisymmetric and TF ripple-induced α-particle losses, again calculated using LOCUST, in two-candidate STEP equilibria with slightly different pedestal parameters. The left-hand plot shows results for the EC-HD FTOP while the right-hand plot was generated for a lower density variant (EC-LD) of this operating point with fewer impurities and a hotter pedestal to illustrate the effect of the pedestal temperature. The fusion power is similar in these two cases. In the left-hand plot, the pedestal top ion temperature is 4.4 keV while in the right-hand plot, it is nearly 7 keV. Switching to a hotter pedestal results in the peak α-particle wall loading increasing by a factor of more than 5 to approximately $1.6\;\text{MW}/{\text{m}}^{\text{2}}$ , despite a slight reduction in the density, while the total loss of α-particle power rises from 1.1 to 2.7 MW (the latter is approximately 0.8% of the α-particle power). It can be seen in figure 6 that the highest power loading occurs on the low field side (LFS) of the main chamber, where the maximum tolerable heat flux is approximately 0.7 MW/m^2^. Moreover, higher losses and power loadings occur when three-dimensional field perturbations are present, in addition to the TF ripple discussed above, and will be further enhanced by ELM control coils, error fields, instabilities and non-axisymmetric ferromagnetic materials. It should be noted that while increasing the pedestal pressure is very beneficial for the fusion performance, the α -particle losses will set a limit to this optimization depending on the actual three-dimensional perturbations as well as the plasma wall gap. Again, as already mentioned in §3.e, the addition of foreseen limiters to the first wall model has no effect on the α -particle losses but need to be repeated when the full three-dimensional wall geometry is available.

A further risk-mitigation strategy with respect to type-I ELMs could be the use of liquid metal armour (LMA) at the strike-point locations or other materials that can withstand the ELM heat load. The readiness level of these technologies is very low and the plasma compatibility of LMA with a power plant-relevant divertor plasma is not well enough understood. Therefore, the default design strategy is an operation without type-I ELMs. Here, the dynamic phases of the plasma (ramp-up and ramp-down) are especially challenging. More details on LMA and the plasma-facing component design are given in the paper ‘Managing the Heat – In-Vessel Components’ in this issue [124].

### (c) Runaway electron mitigation

**Figure 7.**
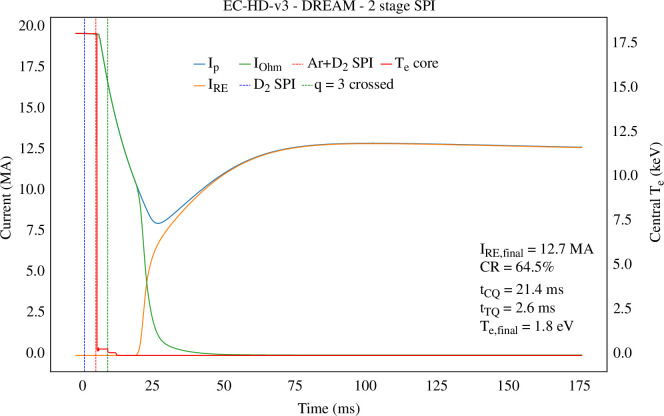
Results from DREAM simulations of a STEP-mitigated disruption, with injection of 2-stage shattered pellets (pure D_2_ at 3 ms, then Ar + D_2_ at 7 ms). The RE beam is mitigated compared with the no-injection case but remains very high, at 12.6 MA for a 1 ms TQ with δB/B=0.2%.

Since the STEP FTOP has a large plasma current $I_{\text{p}}$ (approx. 20 MA), any plasma disruption is likely to induce large EM forces in conducting structures as well as a high current runaway electron (RE) beam. This is mainly owing to the large plasma current, as the avalanche rate of REs scales exponentially with $I_{\text{p}}$ [125]. As in the cases of ITER [126] and SPARC [127], STEP thus falls into the seed-insensitive regime of RE generation, meaning that even small RE seeds, including those from sources that are almost impossible to mitigate (e.g. tritium decay or Compton scattering), can quickly avalanche during disruption current quenches (CQs). This has been confirmed by modelling unmitigated disruptions in STEP with the code DREAM [128], using various assumptions for the thermal quench (TQ). To mitigate this, the STEP concept design includes an extensive set of shattered pellet injectors (SPIs) that follow requirements set by DREAM simulations of SPI-mitigated disruptions [129]. This strategy is largely in line with ITER. One difference with ITER is that high field side (HFS) pure D_2_ pellet lines are planned to make use of so-called plasmoid drifts [130] and reduce the generation of RE seeds pre-CQ (through the hot-tail mechanism). The efficacy of this strategy has been shown for previous STEP concepts [131], but it cannot avoid the generation of a large current RE beam ( >12 MA) when using the latest version of the EC-HD FTOP and the DREAM SPI model [129], see figure 7.

Consequently, the STEP concept design also includes multiple pure D_2_ SPI lines dedicated to RE beam mitigation. Those lines will be used to change the properties of the background plasma during the RE plateau phase, to achieve a benign termination of such a RE beam [132,133]. This method has been successfully applied in several current devices (TCV, ASDEX-Upgrade, DIII-D), including JET [134], and will also be used on ITER. However, owing to the remaining uncertainties and possible limitations of this method (arising from re-avalanching, the upper limit for D_2_ injection), the STEP team is now investigating complementary methods to achieve RE beam mitigation. One of those is to use a passive runaway electron mitigation coil to induce magnetic field perturbations during the CQ that are large enough to counteract the avalanche. This strategy has been successfully modelled for SPARC and DIII-D [127,135,136] and will be tested in those devices in the next few years.

### (d) Key research needs

The most urgent single plasma priority in terms of plugging the gap between present-day STs and STEP is the development of a first principles-based understanding of core confinement in the high-beta regime needed to achieve burning plasma conditions. High priorities for future work in this area, some of which is already in progress, include: (i) building a reduced model to describe transport fluxes from hybrid-KBM turbulence, and exploring the use of machine learning (ML) surrogates to accelerate the evaluation of such models (refer to e.g. [137]); (ii) performing flux-driven simulations using such physics-based models to predict profiles for the STEP FTOPs [46]; (iii) performing the first global gyrokinetic simulations of hybrid-KBM turbulence (which will require the inclusion of compressional magnetic perturbations, $\delta B_{∥}$ , [104] that are usually omitted in global gyrokinetics); (iv) assessing the effects of fusion α-particles and other impurities on microturbulence; (v) improving our understanding of the onset of runaway heat fluxes; (vi) seeking accessible experimental regimes in both current and planned devices where validation may be possible; (vii) studying turbulent transport and the validity of reduced models in the STEP ramp-up. These studies must include the aspects of momentum transport as the turbulence has been found to be very sensitive to plasma rotation. Validation of the flow shear dependence of the hybrid-KBM turbulence poses a challenge, as present-day STs only access high β with substantial torque input that will not be available in STEP. In addition, an understanding of impurity transport in the STEP-relevant regimes (high β , high $q_{\min}>2.3$ , slow rotation) needs to be established including the transport through the pedestal. This is important in particular for W transport. It should be noted that no ST with a high-Z metal wall exists.

Research on no/small ELM regimes in high-performance, DN STs is also key for the development of viable FTOPs for STEP. This will be among the primary future goals of MAST-U and NSTX-U. The former device will also provide a vital opportunity to validate the modelling of EBCD in an ST since it will be equipped with two gyrotrons delivering nearly a megawatt each of microwave power from late 2026 onwards. Accessing high beta regimes in MAST-U and NSTX-U is expected to provide essential data not only on core transport under STEP-like conditions, as noted above, but also on α-particle physics: it is predicted that many of the instabilities driven by energetic ions in present-day devices will be suppressed in STEP, either because of thermal ion Landau damping or the absence of significant fast ion anisotropy [88], but again experimental validation of these predictions is lacking. Finally, MAST-U has been specifically designed to test advanced divertor concepts, and for the development of STEP-relevant scenarios that combine high core plasma performance and radiation with detached exhaust plasma operation. It is clear therefore that MAST-U will play a key role in the STEP project.

More research is also needed on the L- to H-mode transition, which in an ST can be very sensitive to the vicinity of a true CDN configuration with both X-points on the same flux surface [138]. As any DN configuration is necessarily oscillating around CDN into upper and lower SN and in all tokamaks much higher threshold powers are measured in SN with the ion ∇B -drift away from the X-point (lower SN in STEP) the dynamic behaviour of the transition as well as the governing length scales need better understanding. In addition, work to better extrapolate the access conditions to H-mode towards STEP is needed including the impact of momentum input that seems to increase the threshold powers in MAST and MAST-U above current scaling laws [38].

## 5. Conclusions

The following conclusions have emerged from the UKAEA programme to design a first-of-a-kind fusion reactor delivering net electricity, based on the ST concept: (i) a DN configuration is likely to be needed to ensure that exhaust power loads are manageable on the commercial reactor scale; (ii) microwaves (rather than neutral beams or radio-frequency waves) provide the most effective actuator for external HCD; (iii) plasma cross-sections shapes with PT are far preferable to those with NT, owing to unfavourable limits on the normalized plasma pressure (and hence fusion performance) in the latter case; (iv) equilibria with safety factor q>2 across the entire plasma have robust stability properties; (v) the indications so far are that the presence of fusion α-particles need not lead to any difficulties in terms of either unacceptable power loads on plasma-facing components owing to either static three-dimensional magnetic field perturbations or Toroidal Alfvén Eigenmodes; (vi) a complex mitigation scheme will need to be in place to manage the risk of a plasma disruption, even if the control system ensures that such events are extremely rare; (vii) the turbulence regime in the core plasma will be qualitatively different from that in any existing device, and pushes presently available simulation codes to their limit, both in terms of computing resources and physics models; (viii) there are large uncertainties in how to achieve conditions such that confinement is good enough to generate net electricity and in which ELMs are either non-existent or present but manageable. The last two conclusions mean that STEP remains a high-risk project (there are of course other, non-plasma risks, discussed in other papers of this special issue).

We comment finally that several of these conclusions are specific to ST-based reactor concepts, and indeed in some cases underline the inherent advantages of the ST concept. On the other hand, there is a lot of synergy between the STEP work and the solutions needed for power plants based on the conventional aspect ratio tokamak or stellarator concept allowing for a mutual gain. For example, microwave-based external heating and current drive may also turn out to be the best long-term option for power plants. In addition, the need for high plasma current in any tokamak-based reactor means, that while STEP benefits greatly from the work done in support of ITER, novel disruption mitigation schemes of the type being investigated for STEP may also be attractive for future devices.

## Data Availability

To obtain further information on the data and models underlying this paper please contact PublicationsManager@ukaea.uk.
